# State Estimation in Partially Observable Power Systems via Graph Signal Processing Tools

**DOI:** 10.3390/s23031387

**Published:** 2023-01-26

**Authors:** Lital Dabush, Ariel Kroizer, Tirza Routtenberg

**Affiliations:** 1 School of Electrical and Computer Engineering, Ben-Gurion University of the Negev, Beer Sheva 8410501, Israel; 2 Department of Electrical and Computer Engineering, Princeton University, Princeton, NJ 08544, USA

**Keywords:** graph signal processing (GSP), power system state estimation (PSSE), network observability, sensor allocation

## Abstract

This paper considers the problem of estimating the states in an unobservable power system, where the number of measurements is not sufficiently large for conventional state estimation. Existing methods are either based on pseudo-data that is inaccurate or depends on a large amount of data that is unavailable in current systems. This study proposes novel graph signal processing (GSP) methods to overcome the lack of information. To this end, first, the graph smoothness property of the states (i.e., voltages) is validated through empirical and theoretical analysis. Then, the regularized GSP weighted least squares (GSP-WLS) state estimator is developed by utilizing the state smoothness. In addition, a sensor placement strategy that aims to optimize the estimation performance of the GSP-WLS estimator is proposed. Simulation results on the IEEE 118-bus system show that the GSP methods reduce the estimation error magnitude by up to two orders of magnitude compared to existing methods, using only 70 sampled buses, and increase of up to 30% in the probability of bad data detection for the same probability of false alarms in unobservable systems The results conclude that the proposed methods enable an accurate state estimation, even when the system is unobservable, and significantly reduce the required measurement sensors.

## 1. Introduction

Power system state estimation (PSSE) is a critical component of modern energy management systems (EMSs) for multiple purposes, including monitoring, analysis, security, control, and management of the power delivery [1]. The PSSE is conducted using topological information, power measurements, and physical constraints to estimate the voltages (states) at the system buses. The performance and reliability of the PSSE largely depend on the availability and the quality of the measurements [2]. However, there are various realistic scenarios where the system is *partially observable* (also named in the literature as unobservable) ([1] Chpter 4, [3,4,5]), that is, the number of sensors is not sufficiently large, or sensors are not well placed in the network. The observability of the system may be compromised due to communication errors, topology changes, sensor failures  [1], malicious attacks [6,7,8], and electrical blackouts [9]. A direct implication of system unobservability is that conventional estimators that assume deterministic states, such as the commonly used weighted least squares (WLS) estimator, can no longer be used since they are inaccurate, inconsistent, and may have large estimation errors even in the absence of noise [3,10]. Therefore, developing new estimation methods that enable the full functionality of systems without requiring observability is crucial for reliable operation of the power grid.

State estimation in partially observable systems must incorporate additional properties or information beyond the power flow equations in order to obtain a meaningful estimation. Most existing approaches are two-step solutions that first produce additional pseudo-measurements, e.g., based on short-term load forecasting, to make the system observable, and then estimate the states using an existing technique  [11,12,13,14]. However, pseudo-measurements do not contain real-time data, and thus result in an inaccurate estimation. Dynamic state estimation utilizes measurements at different time points [14,15], but needs fast scan rates to capture the dynamics, and is also based on restrictive stationary system assumptions [14]. PSSE that uses data from smart meters and phasor measurement units (PMUs) to overcome observability issues [10,16,17,18] is usually inapplicable, due to the limited deployment of these sensors [19], and their initial installment cost [20,21]. Sparse signal recovery methods [5] use matrix completion to estimate the states under low observability conditions. However, to employ the matrix completion framework, the measurements matrix should be low-rank. Unfortunately, this assumption is system-dependent and does not always hold, due to, e.g., the spatial correlation between loads at neighboring buses. Deep learning techniques have recently been used for pseudo-measurement generation, and for the reconstruction of missing data for PSSE [19,22,23]. However, these techniques heavily depend on the availability of numerous high-quality event labels that are rarely available in practice [24,25]. In addition, some of these methods do not utilize the physical model [19], which may result in poor performance in practice.

Concepts from graph theory and graphical models have been used in power systems for sensor placement [26], topology identification [27,28,29], state estimation [6,30,31], analysis [32,33], and optimal power flow calculation [34,35]. However, the graphical model methods assume a specific statistical structure, which does not necessarily apply in power systems, and often do not use the physical models, which may result in poor performance in practice. GSP is an emerging field that extends concepts and techniques from traditional digital signal processing (DSP) to data on graphs [36,37,38,39]. Recent works have proposed the integration of GSP in power systems, such as the application of the GSP framework for the power grid with PMU data in [40], the spectral graph analysis of power flows in [35], and attack detection by GSP in [7,41,42]. For unobservable power systems, the authors of [43] investigate the use of GSP methods for dynamic state estimation in unobservable systems based on PMUs and advanced metering infrastructures (AMIs) under the assumption of bandlimited graph signals. However, state estimation without full observability that does not depend on PMUs and AMIs using GSP tools has not been demonstrated before. Therefore, using GSP tools has a great potential for overcoming the lack of information in state estimation without full observability.

This research develops new GSP methods for state estimation in power systems based on interpreting the voltage signals (phases and magnitudes) as graph signals. First, it is shown empirically and analytically that the states for static PSSE, i.e., voltages, are smooth graph signals with respect to the nodal admittance matrix, which is a Laplacian matrix in the graph representation of the network. Second, a GSP-WLS estimation method is developed for PSSE in the direct current power flow (DC-PF) model that uses the graph smoothness of the states and does not require the full observability of the network. Next, a new approach for sensor placement is introduced in order to optimize the estimation performance obtained by the GSP-WLS estimator. Finally, the proposed estimation method is extended to the more realistic alternating current power flow (AC-PF) model by developing a regularized Gauss–Newton method for PSSE that uses the smoothness of the voltage phases and magnitudes. The simulations show that the proposed methods can accurately estimate voltage phases and magnitudes, and detect bad data under conditions of low observability, where standard methods cannot (or display poor performance).

The aim of this paper is to establish a GSP framework for state estimation, sensor allocation, and bad data detection in partially observable systems. The key novelties are as follows:

- It is demonstrated that the voltages in the power system can be represented as smooth graph signals, where the graph Laplacian is the admittance matrix. This result can serve as a foundation for developing new GSP tools for other power systems applications in future research.
- New state estimation methods for PSSE in both DC-PF and AC-PF models are developed. These methods use the graph smoothness of the states, and do not require the full observability of the network. While regularization using the Laplacian quadratic form has been applied in various applications, such as image processing [44,45], principal component analysis (PCA) [46], data classification [47,48], and semisupervised learning on graphs [49,50], it has not been conducted before in the context of unobservable power systems. Additionally, the nonlinear measurement equations in the AC-PF model present a new challenge from a GSP perspective, requiring the incorporation of graphical information in the form of Laplacian regularization into the iterative method. As such, these state estimation methods contribute to the expansion of the GSP toolbox for a wide range of applications.
- A new approach for sensor placement is introduced to optimize the estimation performance. As the mean squared error (MSE) of the estimator depends on the unknown state vector, the minimization of the Cram$\overset{´}{e}$r–Rao bound (CRB) is utilized instead. This results in a novel approach that can potentially be applied to other applications in the future.
- Numerical simulations on the IEEE 118-bus system are used to validate the merit of the new estimators and the new sensor placement method under different setups, compared to existing pseudo-measurement and matrix completion techniques.

The rest of this paper is organized as follows. In Section 2, the GSP background is introduced, as well as the model, and the conventional estimation approach. In Section 3, the GSP properties of the states are studied. In Section 4, the GSP-WLS state estimator is derived for the DC-PF model and a sensor placement method. In Section 5, the proposed estimation method to the AC-PF model is extended by deriving the regularized Gauss–Newton method. A simulation study is presented in Section 6, and the discussion and conclusions are provided in Section 8.

## 2. Background and Model

A power system can be represented as an undirected weighted graph, where the nodes and the edges of the graph are the grid buses and transmission lines, respectively. This section begins with background on the theory of GSP in Section 2.1. Then, the considered power flow measurement model, as well as the state estimation and network observability for this model, are presented in Section 2.2.

In the rest of this paper, vectors and matrices are denoted by boldface lowercase letters and boldface uppercase letters, respectively. The notations ${\left(\cdot \right)}^{T}$, ${\left(\cdot \right)}^{-1}$, ${\left(\cdot \right)}^{†}$, and Tr(·) denote the transpose, inverse, Moore–Penrose pseudo-inverse, and trace operators, respectively. The *m*th element of the vector a and the (m,q)th element of the matrix A are denoted by $a_{m}$ and $A_{m,q}$, respectively. Similarly, $A_{S_{1},S_{2}}$ denotes the submatrix of A, the rows and columns of which are indexed by the sets $S_{1}$ and $S_{2}$, where $A_{S}\overset{▵}{=}A_{S,S}$, and $a_{S}$ is a subvector of a containing the elements indexed by S. The cardinality of the set S is denoted by |S|. The gradient of a vector function $g\left(x\right)\in R^{K}$ with respect to $x\in R^{M}$, $\frac{\partial g(x)}{\partial x}$, is a matrix in $R^{K\times M}$, with the (k,m)th element equal to $\frac{\partial g_{k}}{\partial x_{m}}$. The matrices I and 0 denote the identity matrix and the zero matrix with appropriate dimensions, respectively, and ||·|| denotes the Euclidean $l_{2}$-norm.

### 2.1. Background: GSP Framework

Let G(V,ξ) be a general undirected weighted graph, where V={1,…,N} and ξ={1,…,P} are the sets of nodes and edges, respectively. The matrix $W\in R^{N\times N}$ is the weighted adjacency matrix of the graph G(V,ξ), where $W_{k,n}$ denotes the weight of the edge between node *k* and node *n*. It is assumed that $W_{k,n}\geq 0$ and that $W_{k,n}=0$ if no edge exists between *k* and *n*. The graph Laplacian matrix is defined as

(1) $$L_{k,l}=\left\{\begin{array}{cc} \sum_{n=1}^{N}W_{k,n}, & k=l\\ -W_{k,l}, & \mathrm{otherwise} \end{array}\right.,\;\;\;k,l=1,\ldots ,N.$$

The Laplacian matrix is a positive semidefinite matrix with the eigenvalue decomposition

(2) $$L=V\Lambda V^{T},$$

where the columns of V are the eigenvectors of L, $V^{T}=V^{-1}$, and Λ is a diagonal matrix consisting of the ordered eigenvalues of L: $0=\lambda_{1}<\lambda_{2}\leq \ldots \leq \lambda_{N}$. By analogy to signal frequency in DSP, the Laplacian eigenvalues can be interpreted as the graph frequencies that, together with the eigenvectors in V, define the spectrum of the graph G(V,ξ) [37].

A graph signal is a function that assigns a scalar value to each node, and thus, is an *N*-dimensional vector. The graph Fourier transform (GFT) of a graph signal a with respect to the graph G(V,ξ) is [37]

(3) $$\tilde{a}≜V^{-1}a.$$

Similarly, the inverse GFT is obtained by left multiplication of a vector by V. The Dirichlet energy of a graph signal, a, is defined as

(4) $$E_{L}\left(a\right)\overset{▵}{=}a^{T}La=\frac{1}{2}\sum_{k=1}^{N}\sum_{n=1}^{N}W_{k,n}{\left(a_{k}-a_{n}\right)}^{2}=\sum_{k=1}^{N}\lambda_{k}{\tilde{a}}_{k}^{2},$$

where the second equality is obtained by substituting (1), and the last equality is obtained by substituting (2) and (3). The Dirichlet energy is a smoothness measure, which is used to quantify the variability encoded by the graph weights [37]. A graph signal, a, is smooth if

(5) $$E_{L}\left(a\right)\leq \varepsilon ,$$

where ε is small in terms of the specific application [37]. It can be seen that the smoothness condition in (5) requires connected nodes to have similar values (according to (4)), and forces the graph spectrum of the graph signal to be concentrated in the small eigenvalues region (according to (4)).

A graph filter applied on a graph signal is a linear operator that satisfies [51]

(6) $$a_{out}=V\mathrm{diag}\left(\psi \left(\lambda_{1}\right),\cdots ,\psi \left(\lambda_{N}\right)\right)V^{T}a_{in},$$

where $a_{out}$ and $a_{in}$ are the output and input graph signals, diag(a) is a diagonal matrix in which the (n,n)th entry is $a_{n}$, and $\psi (\lambda_{n})$ is the graph filter frequency response at the graph frequency $\lambda_{n}$, n=1,…,N. Low-pass graph filters of order *K* are defined as follows [52].

**Definition 1**. *The graph filter in (6) is a low-pass graph filter of order K with a cutoff frequency at $\lambda_{K}$ if $\eta_{K}<1$, where*

(7) $$\eta_{k}\overset{▵}{=}\frac{\max\{|\psi \left(\lambda_{k+1}\right)|,\ldots ,|\psi \left(\lambda_{N}\right)|\}}{\min\{|\psi \left(\lambda_{1}\right)|,\ldots ,|\psi \left(\lambda_{k}\right)|\}},\;k=1,\ldots ,N-1.$$

This definition implies that if $\eta_{K}<1$, then most of the energy of the graph filter is concentrated in the first *K* frequency bins of the graph filter [52]. Upon passing a graph signal through $V\mathrm{diag}\left(\psi \left(\lambda_{1}\right),\cdots ,\psi \left(\lambda_{N}\right)\right)V^{T}$, the high-frequency components (related to graph frequencies greater than $\lambda_{K}$) are attenuated relative to the low-frequency components (related to graph frequencies lower than $\lambda_{K}$). Accordingly, as long as the input of the filter is a “well-behaved” excitation, and does not possess strong high-pass components, the output signal is a *K*-low-pass graph signal [52], and thus, a *smooth* graph signal for small *K* as defined in (5).

### 2.2. DC-PF Model: State Estimation and Observability

A power system is a network of buses (generators or loads) connected by transmission lines that can be represented as an undirected weighted graph, G(V,ξ), where the set of nodes, V, is the set of *N* buses, and the edge set, ξ, is the set of *P* transmission lines between these buses. The set of all sensor measurements is denoted by M, which includes $M\overset{▵}{=}2P+N$ active power measurements at the *N* buses and at the bi-directional *P* transmission lines. According to the π-model [1], each transmission line, (k,n)∈ξ, which connects buses *k* and *n*, is characterized by an admittance value, $Y_{k,n}$.

The active power and the voltages obey multivariate versions of Kirchhoff’s and Ohm’s laws that result in the nonlinear equations of the AC-PF model (see Section 5). In order to analyze the GSP properties and to simplify the presentation of the new methods, first these equations are approximated using the DC-PF model [1], in which the states are the voltage angles. Therefore, we consider first a DC-PF model with the following noisy measurements of the active power [1]:

(8) z=Hθ+e,

where

- $z={\left[z_{1},\cdots ,z_{M}\right]}^{T}\in R^{M}$ is the active power vector.
- $\theta ={\left[\theta_{1},\cdots ,\theta_{N}\right]}^{T}\in R^{N}$ is the system state vector, where $\theta_{n}$ is the voltage angle at bus *n*. In low-observability systems, it is more convenient to delay the assignment of the reference angle (p. 165 in [2]). Thus, θ includes the angle of the reference bus.
- $e\in R^{M}$ is zero-mean Gaussian noise with covariance R.
- $H\in R^{M\times N}$ is the measurements matrix, which is determined by the topology of the network, the susceptance of the transmission lines, and the meter locations [6]. In particular, the *N* rows of H associated with the meters on the buses that measure the total power flow of the transmission lines connected to these buses together create the nodal admittance matrix B (e.g., see p. 97 in [2]) with the following (k,l)-th element:
  (9) $$B_{k,l}=\left\{\begin{array}{cc} \sum_{n\in N_{k}}-b_{k,n}, & k=l\\ b_{k,l}, & (k,l)\in \xi \\ 0, & \mathrm{otherwise} \end{array}\right.,\;\forall k,l=1,\ldots ,N,$$
  where $N_{k}$ is the set of buses connected to bus *k* and $b_{k,n}<0$ is the susceptance of (k,n)∈ξ, i.e., $b_{k,n}$ equals the imaginary part of $Y_{k,n}$.

The goal of DC-PF PSSE is to recover the state vector, θ, from the measurement vector, z, for various monitoring purposes [1,31]. Since θ also includes the reference bus, without loss of generality, the angle of bus 1 (the reference bus) is set to be $\theta_{1}=0$. The PSSE in this case is implemented using the following WLS estimator [1]:

(10) $$\begin{array}{c} \begin{array}{c} \hat{\theta }{}^{\mathrm{WLS}}=\arg\min_{\theta \in R^{N}}{\left(z-H\theta \right)}^{T}R^{-1}\left(z-H\theta \right)\\ \;\mathrm{such}\;\mathrm{that}\;\theta_{1}=0. \end{array} \end{array}$$

The solution of (10) is

(11) $$\begin{array}{c} \left\{\begin{array}{c} \hat{\theta }{}_{\bar{V}}^{\mathrm{WLS}}=Kz\\ {\hat{\theta }}_{1}^{\mathrm{WLS}}=0 \end{array}\right., \end{array}$$

where

(12) $$K\overset{▵}{=}{\left(H_{M,\bar{V}}^{T}R^{-1}H_{M,\bar{V}}\right)}^{-1}H_{M,\bar{V}}^{T}R^{-1}$$

and $\bar{V}\overset{▵}{=}V\setminus 1$ is the set of all buses except the reference bus.

For state estimation to be feasible, one needs to have enough measurements such that the system state can be uniquely determined by the WLS estimation approach. This observability requirement, before the assignment of reference angles, can be defined by one of the following (p. 165 in [2]).

**Definition 2**. *Assume the DC-PF model from (8). The network is observable if any matrix that is obtained from H by deleting one of its columns has a full column rank of N−1. Alternatively, the network is observable if the following holds: Hθ=0 if, and only if, θ=α1, where α is an arbitrary scalar.*

In particular, since according to Definition 2, $H_{M,\bar{V}}$ has full column rank for an observable system, observability ensures that $H_{M,\bar{V}}^{T}R^{-1}H_{M,\bar{V}}$ is nonsingular, and the WLS estimator from (11) and (12) is well-defined for any observable network. Definition 2 implies the following corollary:

**Corollary 1**. *The network is unobservable if the conditions in Definition 2 are not satisfied.*

In practice, however, network observability is not always guaranteed. In such cases, the WLS estimator from (11) cannot be implemented. Even for observable systems, errors and outliers may have a disastrous effect on the state estimation. In the following, it is shown that incorporating graphical information using GSP tools improves the state estimation performance and enables estimation even in partially observable systems.

## 3. GSP Properties of the States

The power system can be represented as an undirected weighted graph, G(V,ξ), as described at the beginning of Section 2.2. In this context, the state vector, $\theta \in R^{N}$, and the subvector of z from (8) that contains the *N* active power injection measurements at the *N* buses, denoted as $z_{\mathrm{bus}}$, can be interpreted as *graph signals*. In this graph representation, the nodal admittance matrix from (9) is a Laplacian matrix:

(13) L=B.

In this section, the graph low-pass nature and the smoothness of the state vector is established in power systems under normal operation conditions, and where the Laplacian matrix is set to be the nodal admittance matrix, as defined in (13). That is, using the smoothness defined in (4) and (5), we show that

(14) $$E_{L}\left(\theta \right)=\theta^{T}L\theta \leq \varepsilon ,$$

where ε is small relative to the other parameters in the system. These results are consistent with the low-pass graph nature of the complex voltages described in [40]. It can be seen from (14) that the smoothness property depends on the system states and the network topology. The lack of measurements does not change the physical behavior; therefore, the smoothness property is not affected by the system observability.

### 3.1. Theoretical Validation—Output of a Low-Pass Graph Filter

First, we show analytically that the state vector is a low-pass graph signal. By substituting (13) in the model in (8), after taking only the power injection measurements, one obtains

(15) $$\begin{array}{c} z_{\mathrm{bus}}=L\theta +e_{\mathrm{bus}}, \end{array}$$

where $e_{\mathrm{bus}}$ contains the elements of the noise vector, e, that are related to the *N* power measurements at the *N* buses. Equation (15) implies that since L is a Laplacian matrix, it satisfies L1=0 [53], where 1 is a vector of ones with appropriate dimensions. Therefore, the states can be recovered from (15) up to a constant shift, which can be written as

(16) $$\theta =L^{†}\left(z_{\mathrm{bus}}-e_{\mathrm{bus}}\right)+c_{1}1=V\Lambda^{†}V^{T}\left(z_{\mathrm{bus}}-e_{\mathrm{bus}}\right)+c_{1}1,$$

where $c_{1}$ is an arbitrary constant that represents the constant invariant property of the state vector [1], and the last equation is obtained by substituting (2). Without loss of generality, the value of $c_{1}$ is set to be

(17) $$c_{1}=c_{2}1^{T}\left(z_{\mathrm{bus}}-e_{\mathrm{bus}}\right),$$

where $c_{2}$ is an arbitrary constant. Using (17) and the definition of the pseudo-inverse operator, the model in (16) can be written as the following linear graph filter input–output model:

(18) $$\theta =V\mathrm{diag}\left(\psi \left(\lambda_{1}\right),\cdots ,\psi \left(\lambda_{N}\right)\right)V^{T}\left(z_{\mathrm{bus}}-e_{\mathrm{bus}}\right),$$

where

(19) $$\psi \left(\lambda_{n}\right)=\left\{\begin{array}{cc} \frac{1}{\lambda_{n}}, & n=2,\ldots ,N\\ Nc_{2}, & n=1 \end{array}\right..$$

That is, θ is an output of a graph filter with the graph frequency response in (19). This representation holds under the assumption that the network is connected. Therefore, $\lambda_{1}$ is the only zero eigenvalue of L with the eigenvector $\frac{1}{\sqrt{N}}1$ [53].

Since the eigenvalues of L are ordered, $0=\lambda_{1}<\lambda_{2}\leq \lambda_{3}\leq \ldots \leq \lambda_{N}$, it can be seen that the graph frequency response in (19) decreases as *n* increases, as long as $c_{2}>\frac{1}{N\lambda_{2}}$. By substituting (19) in (7), one obtains

(20) $$\eta_{k}=\left\{\begin{array}{cc} \frac{\lambda_{k}}{\lambda_{k+1}}, & 2\leq k\leq N-1\\ \frac{1}{Nc_{2}\lambda_{2}}, & k=1 \end{array}\right.,$$

where $\eta_{k}<1$, k=2,…,N−1. Choosing $c_{2}>>\frac{1}{N\lambda_{2}}$ implies that $\eta_{1}<<1$. Hence, according to Definition 1, $V\mathrm{diag}\left(\psi \left(\lambda_{1}\right),\cdots ,\psi \left(\lambda_{N}\right)\right)V^{T}$ in (18) is a graph low-pass filter of any order K≥1. Since $z_{\mathrm{bus}}$ in (16) includes the generated powers and loads, it can be assumed to be random [54,55], and thus, the input signal, $z_{\mathrm{bus}}-e_{\mathrm{bus}}$, does not possess strong high-pass components. Hence, as explained after Definition 1, the state vector θ is a first-order low-pass graph signal, and a smooth graph signal, as defined in (14).

### 3.2. Experimental Validation in IEEE Systems

**Figure 1:**
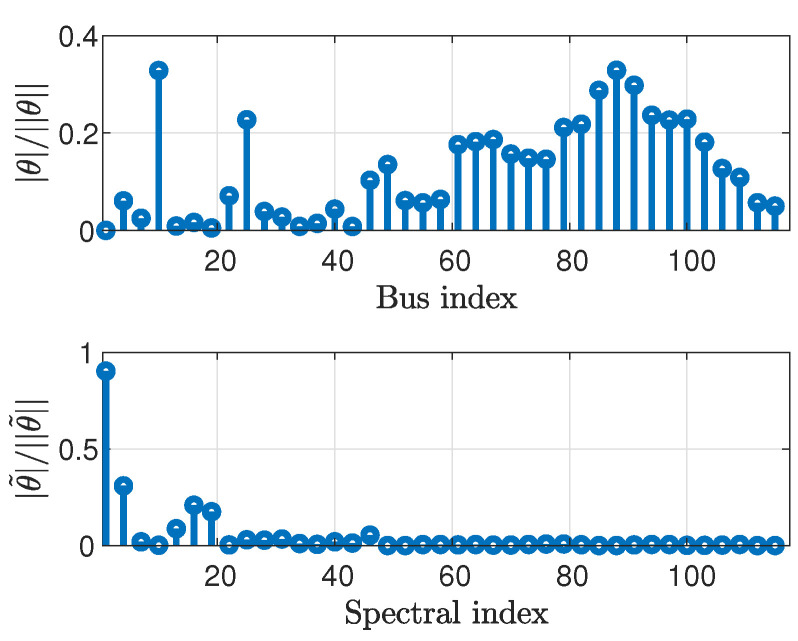
The state vector (**top**) and its GFT (**bottom**) for the IEEE 118-bus system.

**Figure 2:**
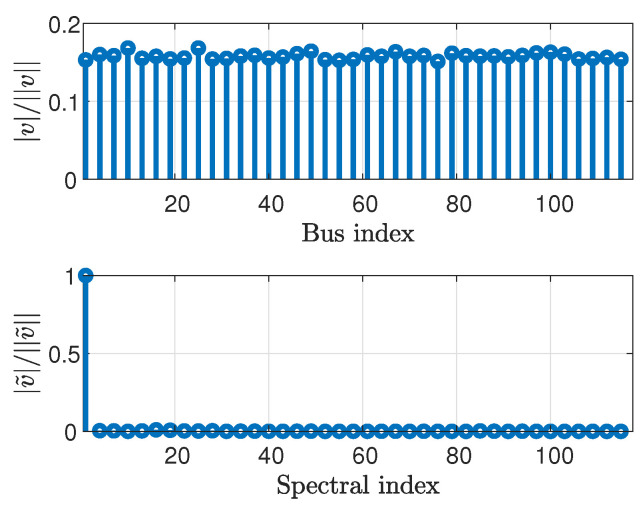
The voltage magnitude vector (**top**) and its GFT (**bottom**) for the IEEE 118-bus system.

**Figure 3:**
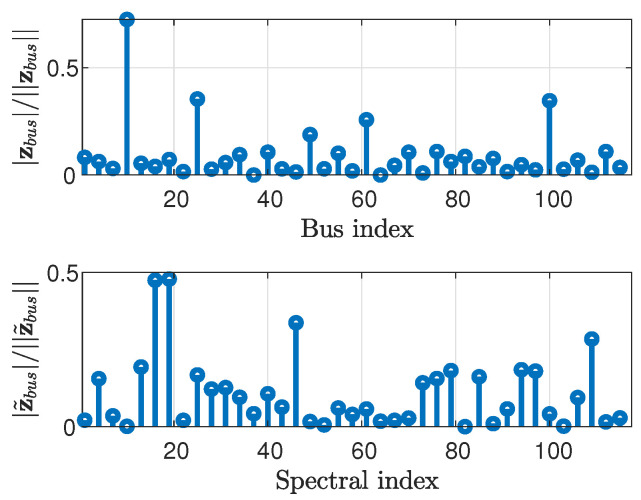
The active power measurement vector (**top**) and its GFT (**bottom**) for the IEEE 118-bus system.

In the following, the smoothness of the state signal is demonstrated in the graph frequency domain for the IEEE test case systems [56]. We also demonstrate the smoothness of the voltage magnitude vector, $v\overset{▵}{=}\left[\right|v_{1}\left|,\cdots ,\right|v_{N}{\left|\right]}^{T}$, which can be interpreted as *graph signals*, which will be used in Section 5. Figure 1 compares the normalized state vector, $\frac{\theta }{\left||\theta |\right|}$, and its GFT (calculated using (3)), $\frac{\tilde{\theta }}{\left|\right|\tilde{\theta }\left|\right|}$, versus bus or spectral indices, for the IEEE 118-bus system [56]. Similarly, Figure 2 presents the normalized voltage magnitude vector, $\frac{v}{\left||v|\right|}$, and its GFT, $\frac{\tilde{v}}{\left|\right|\tilde{v}\left|\right|}$, and Figure 3 presents the normalized power vector, $\frac{z_{\mathrm{bus}}}{\left|\right|z_{\mathrm{bus}}\left|\right|}$, and its GFT, $\frac{{\tilde{z}}_{\mathrm{bus}}}{\left|\right|{\tilde{z}}_{\mathrm{bus}}\left|\right|}$. For the sake of clarity, the vectors in Figure 1, Figure 2 and Figure 3 have been decimated by a factor of 3.

It can be seen that most of the energy of the state signal, i.e., the phases (Figure 1) and the magnitudes (Figure 2) of the voltages, is concentrated in the low graph frequencies region. Accordingly, it can be concluded that the state vector and the voltage magnitude vector are smooth graph signals in the sense of (4). In contrast, the energy of the power injection measurement vector (Figure 3) is uniformly distributed across all graph frequencies. Thus, the power signal cannot be considered to be smooth. Similar results were obtained for other IEEE systems.

Next, we validate experimentally that the states, θ, and the magnitudes, v, are significantly smoother than the power vector, $z_{\mathrm{bus}}$, by comparing their normalized Dirichlet energy for typical IEEE systems, as shown in Table 1. The values of the nodal admittance matrix, B=L, the voltages, and the power data are taken from [56]. It can be seen that the phases and magnitudes are much smoother than the power injection vectors. This result is reasonable, since the phase differences between connected buses are small under normal conditions and the magnitudes are approximately constant [31], while the power may be very different, since each generator/load injects different amounts of power into the system.

## 4. GSP-WLS Estimator in DC-PF Model

The recovery of smooth graph signals by incorporating regularization terms has been well studied in the GSP literature [51,57] and in the context of Laplacian regularization [58,59]. In this section, we cast the state estimation problem as a regularized graph signal recovery problem. In particular, we exploit the smoothness of the state vector, established in Section 3, to develop the smoothness-based regularized GSP-WLS estimator of the states in Section 4.1. The properties of the proposed approach are discussed in Section 4.2, where the main advantage is that it does not require system observability. In Section 4.3, an estimator of the missing power data is introduced as a by-product of this approach. Finally, in Section 4.5, a sensor allocation policy that aims to optimize the performance of the GSP-WLS estimator is designed.

### 4.1. GSP-WLS Estimator for the Partial Measurement Model

In the following, the case where only partial observations of the signal z from (8) are available over a subset of sensors from M is considered, where this subset is denoted by S and S⊆M. A sensor at a particular location provides one row in the measurements matrix, H. Therefore, based on the model in (8), the partial measurement vector can be written as

(21) $$z_{S}=H_{S,V}\theta +e_{S}.$$

Since $e_{S}$ contains the elements of the noise vector, e, of the set of available measurements, S, it is a zero-mean Gaussian noise vector with a covariance matrix $R_{S}$. If the columns of $H_{S,V}$ after deleting one column are linearly dependent, then, from Corollary 1, the new model in (21) with $H_{S,V}$ is not fully observable. In this *partially observable* case, the WLS estimator for the model in (21) cannot be developed via a similar strategy to that in (10) and (11) , since, according to Definition 2, the state, θ, cannot be uniquely (up to a constant) determined from (21).

As a result, we need to incorporate additional properties beyond the power flow equations in (21) to obtain a valid state estimation. Here, we propose to recover θ using the GSP-WLS estimator that incorporates the smoothness constraint from (14). Hence, the GSP-WLS estimator is defined by

(22) $$\begin{array}{c} \;\hat{\theta }{}^{\mathrm{GSP}-\mathrm{WLS}}\;=\;\arg\min_{\theta \in R^{N}}{\left(z_{S}-H_{S,V}\theta \right)}^{T}R_{S}^{-1}\left(z_{S}-H_{S,V}\theta \right)\\ \mathrm{such}\;\mathrm{that}\;1)\;\theta_{1}=0\;\mathrm{and}\;2)\;\theta^{T}L\theta \leq \varepsilon . \end{array}$$

Using $\bar{V}=V\setminus 1$, $\theta_{\bar{V}}$ is the state vector without the reference bus state, $\theta_{1}$, and $L_{\bar{V}}$ is the submatrix of L obtained by removing its first row and column. The smoothness constraint in (14), after substituting $\theta_{1}=0$, can be rewritten as

(23) $$\begin{array}{c} \theta_{\bar{V}}^{T}L_{\bar{V}}\theta_{\bar{V}}\leq \varepsilon . \end{array}$$

Using the smoothness constraint from (23) and substituting $\theta_{1}=0$ in (22), the GSP-WLS estimator is given by

(24) $$\begin{array}{c} \hat{\theta }{}_{\bar{V}}^{\mathrm{GSP}-\mathrm{WLS}}=\arg\min_{\theta_{\bar{V}}\in R^{N-1}}{\left(z_{S}-H_{S,\bar{V}}\theta_{\bar{V}}\right)}^{T}R_{S}^{-1}\left(z_{S}-H_{S,\bar{V}}\theta_{\bar{V}}\right)\\ \mathrm{such}\;\mathrm{that}\;\theta_{\bar{V}}^{T}L_{\bar{V}}\theta_{\bar{V}}\leq \varepsilon ,\; \end{array}$$

and $\hat{\theta }{}_{1}^{\mathrm{GSP}-\mathrm{WLS}}=0$. Then, using the Karush–Kuhn–Tucker (KKT) conditions [60], the minimization problem in (24) can be replaced by the following regularized optimization problem (e.g., see pp. 17–19 in [61]):

(25) $$\begin{array}{c} \hat{\theta }{}_{\bar{V}}^{\mathrm{GSP}-\mathrm{WLS}}=\arg\min_{\theta_{\bar{V}}\in R^{N-1}}\left\{{\left(z_{S}-H_{S,\bar{V}}\theta_{\bar{V}}\right)}^{T}R_{S}^{-1}\left(z_{S}-H_{S,\bar{V}}\theta_{\bar{V}}\right)+\mu \theta_{\bar{V}}^{T}L_{\bar{V}}\theta_{\bar{V}}\right\}, \end{array}$$

and $\hat{\theta }{}_{1}^{\mathrm{GSP}-\mathrm{WLS}}=0$. The term $\theta_{\bar{V}}^{T}L_{\bar{V}}\theta_{\bar{V}}$ is a regularization term, which is based on the smoothness constraint from (23). The parameter μ≥0 is a Lagrange multiplier, which is a tuning parameter that replaces ε and is discussed in Section 4.2. If the system is not fully observable based on the sensors at S, then μ should be larger than zero, i.e., μ>0.

The GSP-WLS estimator from (25) is obtained by equating the derivative of (25) with respect to $\theta_{\bar{V}}$ to zero, which results in [61]

(26) $$\begin{array}{c} \left\{\begin{array}{c} \hat{\theta }{}_{\bar{V}}^{\mathrm{GSP}-\mathrm{WLS}}=\tilde{K}\left(S,\mu \right)z_{S}\\ \hat{\theta }{}_{1}^{\mathrm{GSP}-\mathrm{WLS}}=0 \end{array}\right., \end{array}$$

where

(27) $$\tilde{K}\left(S,\mu \right)\overset{▵}{=}{\left(H_{S,\bar{V}}^{T}R_{S}^{-1}H_{S,\bar{V}}+\mu L_{\bar{V}}\right)}^{-1}H_{S,\bar{V}}^{T}R_{S}^{-1}.$$

For a partially observable system, the matrix $H_{S,\bar{V}}^{T}R_{S}^{-1}H_{S,\bar{V}}$ is a singular matrix and the additional term in (27), and $\mu L_{\bar{V}}$ with μ>0 enables the matrix inversion and improves the numerical stability of the proposed GSP-WLS estimator, since $L_{\bar{V}}$ has full rank (see Lemma 1 in [62]).

### 4.2. Remarks

The main advantage of the proposed GSP-WLS estimator in (26) and (27) is that it does not require full observability of the system. This estimator is a function of the regularization parameter, μ≥0. The determination of μ is discussed in Section 6. More strategies to choose μ are described in the literature (e.g., see Section 1.8 in [61]). The following are special cases of the proposed GSP-WLS estimator:

(1) Full observability: If all sensors are available, then, by substituting S=M and μ=0 in (27) one obtains that
  (28) $$\tilde{K}\left(M,0\right)=K,$$
  where K is defined in (12). Therefore, if S=M, then the GSP-WLS estimator from (26) with μ=0 coincides with the WLS estimator, $\hat{\theta }{}^{\mathrm{WLS}}$, from (11).
(2) Large μ: At the other extreme, for μ→∞, the coefficient matrix from (27) satisfies $\lim_{\mu \to \infty }\tilde{K}\left(S,\mu \right)=0$. Thus, in this case, the GSP-WLS estimator from (26) satisfies $\hat{\theta }{}^{\mathrm{GSP}-\mathrm{WLS}}\to 0$. This zero estimator can be interpreted as the a priori state estimator, which does not use the observations. Thus, taking too large a value of μ is unhelpful.
(3) Relation with the pseudo-measurement WLS (pm-WLS) estimator: The pm-WLS estimator for systems that are not fully observable is based on generating pseudo-measurements of typical power injection/consumption values from historical data [1,24]. In this case, the received measurements are processed together with a priori estimated (predicted) states (without the reference bus), ${\hat{\theta }}_{prior}\in R^{N-1}$, which are assumed to have the error covariance matrix, $R_{prior}\in R^{\left(N-1)\times (N-1\right)}$. The pm-WLS estimator is the maximum a posteriori state estimator [11]:
  (29) $$\begin{array}{c} \left\{\begin{array}{c} {\hat{\theta }}_{\bar{V}}^{\left(\mathrm{pm}-\mathrm{WLS}\right)}=K_{1}z_{S}+K_{2}{\hat{\theta }}_{prior}\\ {\hat{\theta }}_{1}=0 \end{array}\right., \end{array}$$
  where
  (30) $$\begin{array}{ccc} K_{1} & = & {\left(H_{S,\bar{V}}^{T}R_{S}^{-1}H_{S,\bar{V}}+R_{prior}^{-1}\right)}^{-1}H_{S,\bar{V}}^{T}R_{S}^{-1} \end{array}$$
  (31) $$\begin{array}{ccc} K_{2} & = & {\left(H_{S,\bar{V}}^{T}R_{S}^{-1}H_{S,\bar{V}}+R_{prior}^{-1}\right)}^{-1}R_{prior}^{-1}. \end{array}$$
  It can be seen that if ${\hat{\theta }}_{prior}=0$ and $R_{prior}^{-1}=\mu L_{\bar{V}}$, then $K_{1}=\tilde{K}\left(S,\mu \right)$ and the pm-WLS estimator in (29) coincides with the GSP-WLS estimator in (26). Therefore, the proposed GSP-WLS estimator can be interpreted as a special case of the pm-WLS estimator, where the GSP theory provides a mathematical strategy to determine the pseudo-data information. Moreover, in general, the GSP-WLS estimator only requires setting a single scalar parameter, μ, compared with the pm-WLS estimator, which requires setting both $R_{prior}$ and ${\hat{\theta }}_{prior}$.

### 4.3. Estimation of Missing Power Measurements

An important by-product of the GSP-WLS estimator is the following method for reconstructing the missing data of active power measurements. In the partially observable system, we have measurements obtained from the set of sensors, S, which is given by $z_{S}$. Our goal in this subsection is to recover the other measurements that are included in the vector $z_{M\setminus S}$. Based on the model in (8) (similar to (21)), the measurement vector of the partially observable system can be written as

(32) $$z_{M\setminus S}=H_{M\setminus S,V}\theta +e_{M\setminus S},$$

where $e_{M\setminus S}$ is a zero-mean noise vector with a covariance matrix $R_{M\setminus S}$. By substituting the GSP-WLS estimator from (26) in (32) and removing the noise term, the following WLS-type estimator of the missing power measurements is obtained:

(33) $${\hat{z}}_{M\setminus S}=H_{M\setminus S,V}\hat{\theta }{}^{\mathrm{GSP}-\mathrm{WLS}}.$$

By recovering the lost power data, the EMS can also monitor the unobservable part of the system [63].

### 4.4. Detection of Bad Data in Unobservable Systems

State estimators can also be used for bad data detection by plugging it into any detector that is based on a state estimator. In particular, in this paper, the following bad data detection methods that are based on a general estimator $\hat{\theta }$ can be used with the new estimator:

- Largest normalized residual test (LNR) [1]:
  (34) $$\frac{1}{\sigma^{2}}{∥z_{S}-H_{\nu ,S}\hat{\theta }∥}_{\infty }\;\overset{H_{1}}{\underset{H_{0}}{≷}}\;\tau ,$$
  where the infinity norm of a vector a is defined by ${∥a∥}_{\infty }=\max_{i}\left|a_{i}\right|$.
- J(θ) test with $R=\sigma^{2}I$ [1]:
  (35) $$J\left(\hat{\theta }\right)=\frac{1}{\sigma^{2}}{\left(z_{S}-H_{\nu ,S}\hat{\theta }\right)}^{T}\left(z_{S}-H_{\nu ,S}\hat{\theta }\right)\;\overset{H_{1}}{\underset{H_{0}}{≷}}\;\tau .$$
- The GFT-based detector from [7] that was developed for the detection of false data injection (FDI) attacks. The GFT-based detection scheme calculates the GFT of an estimated grid state, $\hat{\theta }$, and filters the graph’s high-frequency components. By comparing the maximum norm of this outcome with a threshold, it can detect the presence of FDI attacks.

In both (34) and (35), τ denotes a chosen threshold, and $H_{0}$ and $H_{1}$ denote the hypotheses of good/bad data, respectively.

Then, the proposed estimator from (26) can be used for bad data detection by plugging it into the detectors that are based on a state estimator; that is, by substituting $\hat{\theta }={\hat{\theta }}^{\mathrm{GSP}-\mathrm{WLS}}$ in the LNR test from (34), the J(θ) test from (35), and the GFT-based detector from [7].

### 4.5. Optimization of the Sampling Policy

Sensor locations have a significant impact on the estimation performance of power systems [64]. Therefore, in this subsection, we design a sensor allocation policy for the model in (21) that aims to minimize the MSE of the GSP-WLS estimator, $\mathrm{MSE}\left(\hat{\theta }\right)=E\left[{\left(\hat{\theta }-\theta \right)}^{T}\left(\hat{\theta }-\theta \right)\right]$. However, it can be shown that the MSE of $\hat{\theta }{}^{\mathrm{GSP}-\mathrm{WLS}}$ is a function of the unknown state vector, θ, and thus, cannot be used as an objective function for the optimization of the sensor locations. Therefore, the MSE is replaced by the CRB [65], which is a lower bound on the MSE.

In this subsection, we treat the MSE, bias, and CRB of the vector $\theta_{\bar{V}}$ (without the reference bus for the sake of simplicity). By substituting $\theta_{1}=0$ in the model in (21), one obtains that the partial measurement vector obtained from a sensor subset S is a Gaussian vector with mean $H_{S,\bar{V}}\theta_{\bar{V}}$ and covariance $R_{S}$:

(36) $$\begin{array}{c} z_{S}∼N\left(H_{S,\bar{V}}\theta_{\bar{V}},R_{S}\right). \end{array}$$

The CRB for this Gaussian vector, which is a lower bound on the MSE, is given by (pp. 45–46 in [65])

(37) $$\mathrm{CRB}\left(S\right)\overset{▵}{=}\mathrm{Tr}\left(\left(I+\frac{\partial b(S)}{\partial \theta_{\bar{V}}}\right){\left(H_{S,\bar{V}}^{T}R_{S}^{-1}H_{S,\bar{V}}\right)}^{†}{\left(I+\frac{\partial b(S)}{\partial \theta_{\bar{V}}}\right)}^{T}\right),$$

where $b\left(S\right)\overset{▵}{=}E\left[{\hat{\theta }}_{\bar{V}}-\theta_{\bar{V}}\right]$ is the bias of the estimator and $\frac{\partial b(S)}{\partial \theta_{\bar{V}}}$ is its gradient. Using the model in (21) and the estimator in (26), it can be seen that the bias of the GSP-WLS estimator is

(38) $$\begin{array}{c} b\left(S\right)=E\left[{\hat{\theta }}_{\bar{V}}^{\mathrm{GSP}-\mathrm{WLS}}-\theta_{\bar{V}}\right]=\tilde{K}\left(S,\mu \right)H_{S,\bar{V}}\theta_{\bar{V}}-\theta_{\bar{V}}, \end{array}$$

where $\tilde{K}\left(S,\mu \right)$ is defined in (27). Accordingly, the gradient of (38) with respect to $\theta_{\bar{V}}$ is

(39) $$\begin{array}{c} \frac{\partial b(S)}{\partial \theta_{\bar{V}}}=\tilde{K}\left(S,\mu \right)H_{S,\bar{V}}-I. \end{array}$$

By substituting (39) in (37), we obtain that the CRB on the MSE of estimators with the GSP-WLS bias is given by

(40) $$\mathrm{CRB}\left(S\right)=\mathrm{Tr}\left(\tilde{K}\left(S,\mu \right)H_{S,\bar{V}}{\left(H_{S,\bar{V}}^{T}R_{S}^{-1}H_{S,\bar{V}}\right)}^{†}H_{S,\bar{V}}^{T}{\tilde{K}}^{T}\left(S,\mu \right)\right).$$

By substituting (27) in (40) and using the pseudo-inverse property, $A=AA^{†}A$, one obtains

(41) $$\mathrm{CRB}\left(S\right)=\mathrm{Tr}\left(\tilde{K}\left(S,\mu \right)R_{S}{\tilde{K}}^{T}\left(S,\mu \right)\right).$$

The CRB in (41) is not a function of the unknown state vector, θ, and thus can be used as an optimization criterion for choosing the sensor locations. We assume a constrained quantity of sensing resources, e.g., due to a limited energy and communication budget. Therefore, the problem of the selection of sensor locations with only $\tilde{q}$ sensors can be written as follows:

(42) $$S^{opt}=\arg\min_{S\subset M}\mathrm{CRB}\left(S\right)\;s.t.\;|S|=\tilde{q}=\arg\min_{S\subset M}\left(\tilde{K}\left(S,\mu \right)R_{S}{\tilde{K}}^{T}\left(S,\mu \right)\right)\;s.t.\;\left|S\right|=\tilde{q},$$

where the last equality is obtained by substituting (41).

**Figure 4:**
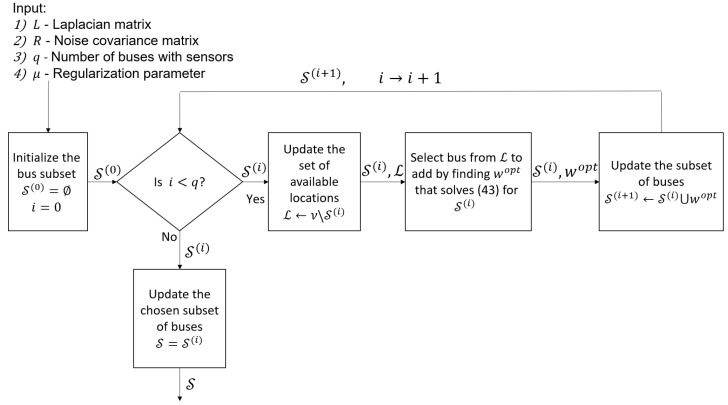
Flow of the proposed GSP greedy selection of the measured buses (Algorithm 1).

It is assumed that in the measured buses, all the relevant power measurements are given. Thus, S is uniquely determined by the buses chosen for the measurements. For the sake of simplicity, in the optimization approach, we take $H_{V,\bar{V}}=L_{V,\bar{V}}$ and replace the selection of $\tilde{q}$ sensors by the selection of *q* buses. Therefore, by substituting $H_{V,\bar{V}}=L_{V,\bar{V}}$, the problem in (42) is replaced by the problem of selecting the optimal buses in the CRB sense. However, finding the set of *q* locations among all the *N* buses with the smallest CRB is a combinatorial optimization with a computational complexity of Nq, which is practically infeasible. Therefore, we propose a greedy algorithm, Algorithm 1, for the practical implementation of the sampling scheme. The idea behind this algorithm is to iteratively add to the sampling set those buses that lead to the minimal CRB. In addition, Figure 4 illustrates the data flow diagram of this greedy algorithm for selecting the measured buses.
**Algorithm 1** Greedy selection of the measured buses**Input:**(1)  Laplacian matrix, L, and noise covariance matrix, R(2)  Number of buses with sensors, *q*(3)  Regularization parameter, μ**Output:** Subset of *q* buses, S
1:Initialize the bus subset $S^{\left(0\right)}=\emptyset$ and the iteration, i=02:**while**i<q**do**3:   Update the set of available locations, $L=V\setminus S^{\left(i\right)}$4:   Find the optimal bus to add:
(43)$$w^{opt}=\arg\min_{w\in L}\mathrm{Tr}\left(\left\{\tilde{K}\left(S^{\left(i\right)}\cup w\right\},\mu \right)R_{\left(S^{\left(i\right)}\cup w\right)}{\tilde{K}}^{T}\left(\left\{S^{\left(i\right)}\cup w\right\},\mu \right)\right),$$
where $\tilde{K}$ is defined in (27) with $H_{V,\bar{V}}=L_{V,\bar{V}}$5:   Update the subset of buses, $S^{\left(i+1\right)}\leftarrow S^{\left(i\right)}\cup w^{opt}$, and the iteration, i←i+16:**end while**7:Update the chosen subset of buses: $S=S^{\left(i\right)}$


## 5. Extension to the AC-PF Model

Since the problem of low observability mainly occurs in distribution systems, which requires AC state estimation, in this section, the GSP-WLS estimator is extended to the AC-PF model, where voltage magnitudes are estimated as well. In particular, the conventional PSSE in the AC-PF model is described in Section 5.1. Then, in Section 5.2, the proposed iterative regularized Gauss–Newton method that exploits the smoothness property of the voltage phases and magnitudes in each iteration is presented. In Section 5.3, the properties of the proposed GSP Gauss–Newton algorithm are discussed.

### 5.1. Model, State Estimation, and Observability

In the following, we replace the DC-PF model from (8) by the following nonlinear AC-PF model equations:

(44) z=h(x)+e,

where

- $z\in R^{M}$ is the measurement vector that includes the active and reactive branch power flows and power injections.
- h(x) is the measurement function, which is determined by the sensor types and their locations in the network.
- $x=[\theta_{2},\cdots ,\theta_{N},|v_{2}\left|,\cdots ,\right|v_{N}{\left|\right]}^{T}\in R^{2N-2}$, is the state vector here, where bus 1 is the reference bus, and thus, $\theta_{1}=0$ and $v_{1}$ is known (e.g., see Chapter 4 in [1]).

The specific forms and parameters of (44) with different levels of modeling details can be found, e.g., in Chapter 2 of [1].

Similar to the WLS estimator for the DC-PF model in (10), the AC-PF state estimator is usually based on minimizing the following WLS objective function:

(45) $$\begin{array}{c} J\left(z,h\left(x\right),R\right)={\left(z-h\left(x\right)\right)}^{T}R^{-1}\left(z-h\left(x\right)\right), \end{array}$$

with respect to x [1]. The first-order optimality condition for the unconstrained minimization problem in (45) is given by

(46) $$\begin{array}{c} g\left(x\right)\overset{▵}{=}\frac{\partial J(z,h(x),R)}{\partial x}=-H^{T}\left(x\right)R^{-1}\left(z-h\left(x\right)\right)=0, \end{array}$$

where $H\left(x\right)\overset{▵}{=}\frac{\partial h(x)}{\partial x}$ is the Jacobian matrix of h(x) at x. Solving the nonlinear equation in (46) using the Gauss–Newton method [1,2] results in the following iterative system:

(47) $$x^{\left(i+1\right)}=x^{\left(i\right)}+G^{-1}\left(x^{\left(i\right)}\right)H^{T}\left(x^{\left(i\right)}\right)R^{-1}\left(z-h\left(x^{\left(i\right)}\right)\right),$$

where $x^{\left(i\right)}$ is the state estimator at the *i*th iteration and

(48) $$\begin{array}{c} G\left(x\right)=H^{T}\left(x\right)R^{-1}H\left(x\right) \end{array}$$

is the gain matrix. Iterating until convergence, i.e., until $\left|\right|x^{\left(i+1\right)}-x^{\left(i\right)}\left|\right|\leq \delta$, one will obtain the solution of PSSE.

The observability requirement for the AC-PF model can be defined as follows (see Chapter 4.6 in [1], Donti et al. [5]).

**Definition 3**. *Assume the AC-PF model from (44). The network is observable if G(x) is a nonsingular matrix for any x in the solution space.*

By observing (48), it can be seen that if H(x) has a full column rank of 2N−2, then the network is observable in the AC-PF sense. This observability condition should be satisfied in each iteration of the Gauss–Newton iterative algorithm.

### 5.2. GSP-Based Gauss–Newton Algorithm

Similar to Section 4, here we consider the case where only partial observations of z from (44) are available over a subset of sensors S⊆M. That is, based on the model in (44), the partial measurement AC-PF model can be written as

(49) $$\begin{array}{c} z_{S}=h_{S}\left(x\right)+e_{S}, \end{array}$$

where $e_{S}$ is a zero-mean Gaussian noise vector with a covariance matrix $R_{S}$, as in (21). The Jacobian matrix of the model in (49) is $H_{S,\bar{\bar{V}}}\left(x\right)=\frac{\partial h_{S}\left(x\right)}{\partial x}$, where $\bar{\bar{V}}$ indicates the set of all the columns in H(x). If the columns of $H_{S,\bar{\bar{V}}}\left(x\right)$ are linearly dependent, then G(x) is a singular matrix, and from Definition 3, the new system in (49) is not fully observable. In this case, the Gauss–Newton iterative procedure for the minimization of (45) cannot be implemented, since the update of the solution cannot be uniquely determined from (47).

In order to tackle this problem, we incorporate the smoothness constraints from (23) and from (5) with a=v. Hence, the GSP-WLS estimator for the AC-PF model is defined by

(50) $$\begin{array}{c} \hat{x}{}^{\mathrm{GSP}-\mathrm{WLS}}=\arg\min_{x={\left[\theta_{\bar{V}}^{T},v^{T}\right]}^{T}\in R^{2N-2}}J\left(z_{S},h_{S}\left(x\right),R_{S}\right)\\ \mathrm{such}\;\mathrm{that}\;1)\;\theta_{\bar{V}}^{T}L_{\bar{V}}\theta_{\bar{V}}\leq \varepsilon_{\theta }\;\\ \;\mathrm{and}\;2)\;{\left(v_{\bar{V}}-v_{1}1\right)}^{T}L_{\bar{V}}\left(v_{\bar{V}}-v_{1}1\right)\leq \varepsilon_{v}, \end{array}$$

where the function *J* is defined in (45), and $\varepsilon_{\theta },\varepsilon_{v}$ are the tuning parameters of the smoothness of θ and v.

Using the KKT conditions [60], the minimization in (50) can be replaced by the following regularized optimization:

(51) $$\begin{array}{c} \hat{x}{}^{\mathrm{GSP}-\mathrm{WLS}}=\;\arg\min_{x\in R^{2N-2}}J_{\mathrm{reg}}\left(z_{S},h_{S}\left(x\right),R_{S},\bar{L}\left(\mu_{\theta },\mu_{v}\right)\right), \end{array}$$

where

(52) $$J_{\mathrm{reg}}\left(z,h\left(x\right),R,\bar{L}\left(\mu_{\theta },\mu_{v}\right)\right)\overset{▵}{=}J\left(z,h\left(x\right),R\right)+{\left(x-x_{0}\right)}^{T}\bar{L}\left(\mu_{\theta },\mu_{v}\right)\left(x-x_{0}\right),$$

(53) $$\bar{L}\left(\mu_{\theta },\mu_{v}\right)\overset{▵}{=}\left[\begin{array}{cc} \mu_{\theta }L_{\bar{\nu }} & 0\\ 0 & \mu_{v}L_{\bar{\nu }} \end{array}\right],$$

and $x_{0}\overset{▵}{=}{\left[0^{T},v_{1}1^{T}\right]}^{T}$. The right term in (52) is a regularization term, which is based on the smoothness property of the phases and magnitudes of the voltages, established in Section 3. The parameters $\mu_{\theta },\mu_{v}\geq 0$ are Lagrange multipliers that replace $\varepsilon_{\theta },\varepsilon_{v}$ as regularization parameters, and their tuning is discussed in Section 5.3 and Section 6.3.

The minimum of the quadratic objective function, $J_{\mathrm{reg}}\left(z_{S},h_{S}\left(x\right),R_{S},\bar{L}\left(\mu_{\theta },\mu_{v}\right)\right)$, with respect to x can be determined using the first order optimality conditions as follows:

(54) $$g_{\mathrm{reg}}\left(x\right)\overset{▵}{=}\frac{\partial J_{\mathrm{reg}}\left(z_{S},h_{S}\left(x\right),R_{S},\bar{L}\left(\mu_{\theta },\mu_{v}\right)\right)}{\partial x}=-H_{S,\bar{\bar{V}}}^{T}\left(x\right)R_{S}^{-1}\left(z_{S}-h_{S}\left(x\right)\right)+\bar{L}\left(\mu_{\theta },\mu_{v}\right)\left(x-x_{0}\right)=0.$$

**Figure 5:**
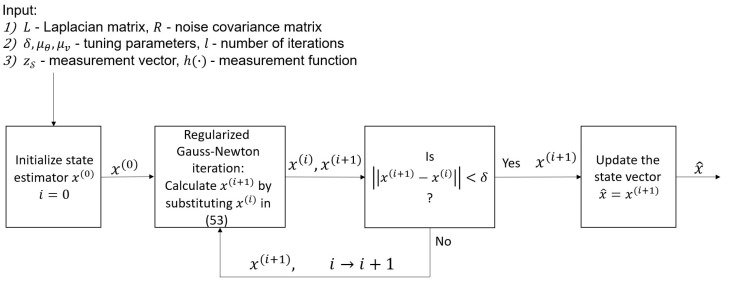
Flow of the proposed regularized Gauss–Newton scheme.

Then, similar to (47), the nonlinear equation, $g_{\mathrm{reg}}\left(x\right)=0$, is solved using the following Gauss–Newton method iteration:

(55) $$x^{\left(i+1\right)}=x^{\left(i\right)}+G_{\mathrm{reg}}^{-1}\left(x^{\left(i\right)}\right)\left(H_{\bar{\bar{V}},S}^{T}\left(x^{\left(i\right)}\right)R_{S}^{-1}\left(z_{S}-h_{S}\left(x^{\left(i\right)}\right)\right)-\bar{L}\left(\mu_{\theta },\mu_{v}\right)\left(x^{\left(i\right)}-x_{0}\right)\right),$$

where

(56) $$\begin{array}{c} G_{\mathrm{reg}}\left(x\right)\overset{▵}{=}\frac{\partial g_{\mathrm{reg}}\left(x\right)}{\partial x}=H_{S,\bar{\bar{V}}}^{T}\left(x\right)R_{S}^{-1}H_{S,\bar{\bar{V}}}\left(x\right)+\bar{L}\left(\mu_{\theta },\mu_{v}\right) \end{array}$$

is the new gain matrix. Solving this equation and iterating until the required accuracy, δ, is reached, i.e., $\left|\right|x^{\left(i+1\right)}-x^{\left(i\right)}\left|\right|\leq \delta$, one will obtain the proposed GSP-WLS estimator for the AC-PF model. It can be seen that for a partially observable system, $H_{S,\bar{\bar{V}}}^{T}\left(x\right)R_{S}^{-1}H_{S,\bar{\bar{V}}}\left(x\right)$ is a singular matrix, and the additional terms in (56), $\bar{L}\left(\mu_{\theta },\mu_{v}\right)$ from (53) with $\mu_{\theta }>0$, and/or $\mu_{v}>0$, can enable the matrix inversion of $G_{\mathrm{reg}}\left(x\right)$ and improve the numerical stability of the GSP-WLS estimator for the AC-PF model. The iterative solution is summarized in Algorithm 2. For the initialization, we suggest that one use the “flat start”, where all bus voltages are 1 per unit and have the same phase [1]. Figure 5 presents the flow of the iterative regularized Gauss–Newton algorithm through a data flow diagram.
**Algorithm 2:** Regularized Gauss–Newton (GSP-WLS)**Input:**(1)  Laplacian matrix, L, and noise covariance matrix, $R_{S}$(2)  Tuning parameters: δ, $\mu_{\theta }$, $\mu_{v}$ and number of iterations, *l*(3)  Measurement vector, $z_{S}$, and the function, $h_{S}\left(\cdot \right)$**Output:** State estimator, $\hat{x}$1:Initialize the state vector $x^{\left(0\right)}$2:**for**i=0,⋯,l**do**3:   Calculate the right hand side of (55) for $x^{\left(i\right)}$4:   Solve (55) for $x^{\left(i+1\right)}$5:   **if** $\left|\right|x^{\left(i+1\right)}-x^{\left(i\right)}\left|\right|<\delta$: **break**6:**end for**7:Update the state vector: $\hat{x}=x^{\left(i+1\right)}$

### 5.3. Remarks

In the following, we present special cases of the proposed GSP-WLS estimator for the AC-PF model implemented by the regularized Gauss–Newton method.

(1) Full observability: If the information from all sensors is available, i.e., if $h_{S}\left(x\right)=h\left(x\right)$, then, by substituting $\mu_{\theta }=\mu_{v}=0$ in (55) one obtains that $G_{\mathrm{reg}}\left(x\right)=G\left(x\right)$, where G(x) and $G_{\mathrm{reg}}\left(x\right)$ are defined in (48) and (56), respectively. Accordingly, in this case, the Gauss–Newton iteration of the GSP-WLS estimator in (55) coincides with the Gauss–Newton iteration in (47).

(2) Relation with the pm-WLS estimator: Similar to the case of the DC-PF model, the pm-WLS estimator for partially observable systems is calculated based on the measurements and the a priori estimated (predicted) states, ${\hat{x}}_{prior}\in R^{2N-2}$, with the forecasting error covariance matrix, $R_{prior}$. As a result, the following pm-WLS estimator is obtained [11]:

(57) $$\hat{x}{}^{\mathrm{pm}-\mathrm{WLS}}=\arg\min_{x\in R^{2N-2}}\left\{J\left(z_{S},h_{S}\left(x\right),R_{S}\right)+{\left(x-{\hat{x}}_{prior}\right)}^{T}R_{prior}^{-1}\left(x-{\hat{x}}_{prior}\right)\right\}.$$

The pm-WLS estimator for the AC-PF model can be calculated with the Gauss–Newton method [11]. It can be seen that if we substitute ${\hat{x}}_{prior}=x_{0}$ and $R_{prior}^{-1}=\bar{L}\left(\mu_{\theta },\mu_{v}\right)$, then the pm-WLS estimator from (57) coincides with the GSP-WLS estimator from (51) and (52). Hence, similar to the DC-PF model, the proposed GSP-WLS estimator can be interpreted as a special case of the pm-WLS estimator, where the GSP theory provides the mathematical justification for the determination of the pseudo-data information.

## 6. Results

In this section, the performance of the proposed methods is compared with that of existing methods. In Section 6.2, the performance of the GSP-WLS estimator from Section 4 is evaluated. In Section 6.3, the performance of the regularized Gauss–Newton method from Section 5 is investigated. The influence of the sampling policy from Section 4.5 is examined for both cases. In Section 6.4, the use of the new estimator for bad data detection is demonstrated.

### 6.1. Simulations Platform and Parameters

All the simulations were performed with Matlab R2020b. The measurements were generated according to the AC-PF model from (44) with h(·)

(58) $$\begin{array}{c} h_{2n-1}\left(x\right)=\sum_{k=1}^{N}|v_{n}\left|\right|v_{k}|\left(G_{n,k}\cos\left(\theta_{n}-\theta_{k}\right)+B_{n,k}\sin\left(\theta_{n}-\theta_{k}\right)\right),\;\;\;n=1,\ldots ,N,\\ h_{2n}\left(x\right)=\sum_{k=1}^{N}|v_{n}\left|\right|v_{k}|\left(G_{n,k}\sin\left(\theta_{n}-\theta_{k}\right)-B_{n,k}\cos\left(\theta_{n}-\theta_{k}\right)\right),\;\;\;n=1,\ldots ,N, \end{array}$$

for the real and reactive power injection measurement at bus *n* and

(59) $$\begin{array}{c} h_{2N+2f(n,k)-1}\left(x\right)=|v_{n}{|}^{2}G_{n,n}-|v_{k}\left|\right|v_{n}|\left(G_{n,k}\cos\left(\theta_{n}-\theta_{k}\right)+B_{n,k}\sin\left(\theta_{n}-\theta_{k}\right)\right),\;\;\;\forall n\in N_{k},\;\;\;k=1,\ldots ,N,\\ h_{2N+2f(n,k)}\left(x\right)=-|v_{n}{|}^{2}B_{n,n}-|v_{n}\left|\right|v_{k}|\left(G_{n,k}\sin\left(\theta_{n}-\theta_{k}\right)-B_{n,k}\cos\left(\theta_{n}-\theta_{k}\right)\right),\;\;\;\forall n\in N_{k},\;\;\;k=1,\ldots ,N, \end{array}$$

for the real and reactive power flow from bus *n* to bus *k* measurements (f(n,k) is a one-to-one mapping of all (n,k)∈ξ to [1,2P]), where $x=[\theta_{2},\cdots ,\theta_{N},|v_{2}\left|,\cdots ,\right|v_{N}{\left|\right]}^{T}\in R^{2N-2}$ is the state vector here, bus 1 is the reference bus, and thus $\theta_{1}=0$ and $v_{1}$ is known. The values of the conductance and the susceptance matrices, B and G, as well as the values of the voltage angles and magnitudes, were taken from the IEEE 118-bus test case recorded in [56]. This IEEE 118-bus test case represents a simple approximation of the American electric power system (in the U.S. Midwest) as of December 1962. This IEEE 118-bus system, which has N=118 buses and, at most, M=952 power measurements, contains 19 generators, 35 synchronous condensers, 177 lines, 9 transformers, and 91 loads. Bus number 1 is set to be the slack bus. Gaussian white noise was added to generate several measurements from the recorded grid state. In particular, in the simulations described in the following subsections, the noise covariance matrix is set to $R=\sigma^{2}I$, where, unless otherwise stated, $\sigma^{2}=0.01$. The performance is evaluated using 1000 Monte Carlo simulations.

We compare the estimation performance of the different estimators implemented for the following bus selection policies:

(i) Random bus selection policy (rand.)—the measured buses are randomly chosen independently from {1,…,N}, where for more than 72 buses only observable systems are taken.
(ii) Experimentally designed sampling (E-design) [38]—the buses are chosen to maximize the smallest singular value of the matrix $V_{S,\{1,\ldots ,R\}}$, where *R* is set to 48. The basic assumption behind this method (which was suggested in [66] for power systems) is that the measured graph signal (here, the power signal) is an *R*-bandlimited signal in the graph frequency domain. That is, the GFT of $z_{\mathrm{bus}}$ satisfies ${\left({\tilde{z}}_{\mathrm{bus}}\right)}_{n}=0$, n=R+1,…,N, where *R* is the cutoff frequency. As can be seen in Figure 3, in practice, the *R*-bandlimitness assumption does not hold for the power signal.
(iii) Minimum CRB (Algorithm 1)—the proposed bus selection policy from Algorithm 1.

It should be noted that the CRB from (37) is not presented in the following simulations, since it is not the main goal of this study, and in order to increase the interpretability of the figures.

**Figure 6:**
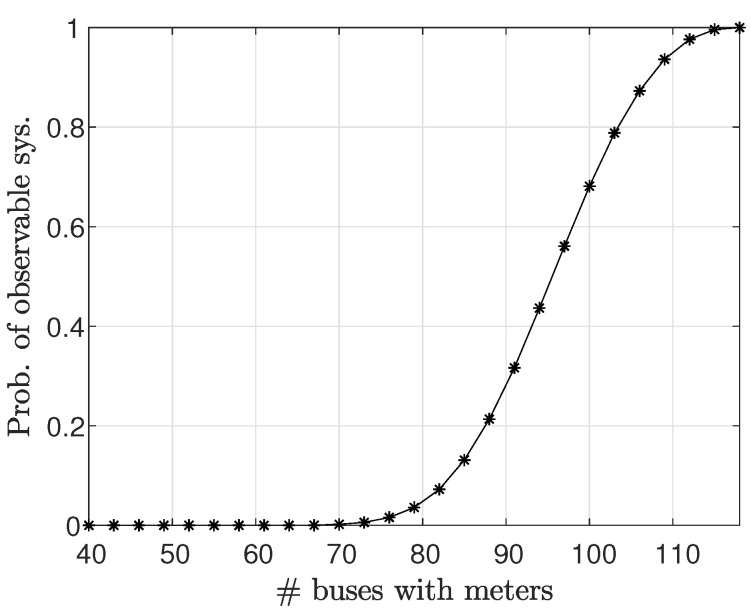
The estimated probability for the IEEE 118-bus system to be observable versus the number of measured buses.

Figure 6 presents the estimated probability for the system to be observable, according to Definition 2, versus the number of measured buses. The estimated probability of observability is calculated as the percentage of scenarios with observable systems in 100,000 Monte Carlo simulations for randomly selected buses in the system. It can be seen that this probability of observability increases as the number of measured buses increases, and that for fewer than 72 measured buses, the IEEE 118-bus system will not be observable with probability 1.

### 6.2. State Estimation and Sampling under the DC-PF Model

In this subsection, we evaluate the performance of the following estimators:

1. The pm-WLS estimator from [11], generated with $R_{prior}^{-1}=0.5I$, and where ${\hat{\theta }}_{prior}$ is randomly chosen from a zero-mean Gaussian distribution with covariance 0.015I.
2. The matrix compilation (mc) method [5], implemented in this subsection by substituting the DC model from (8) in the constraints of the method in Equations (8) and (12) in [5].
3. The proposed GSP-WLS estimator (26) and (27) with μ=0.1.

**Figure 7:**
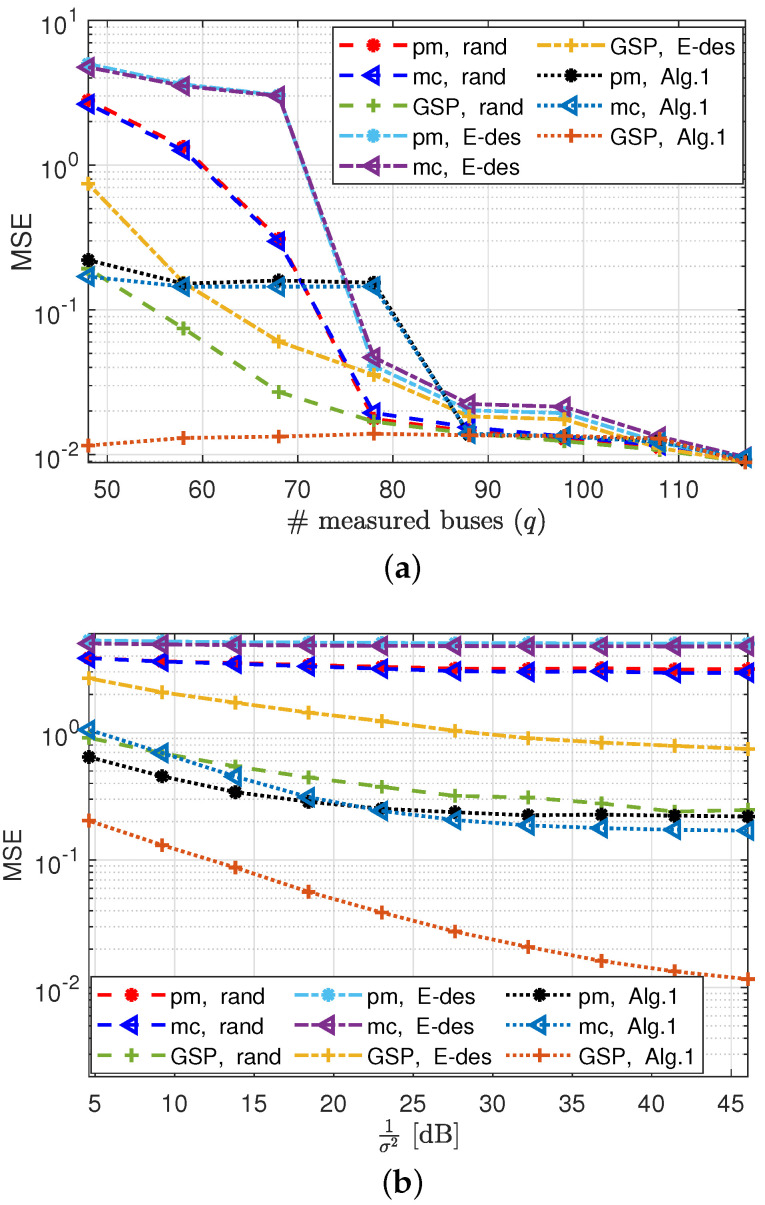
State estimation under the DC−PF model: the MSE of the GSP−WLS from (26) and (27), pm−WLS [11], and mc [5] estimators for random, E−design [38], and Algorithm 1 bus selection policies versus (**a**) the number of buses, *q*, with $\sigma^{2}=0.01$, and (**b**) $\frac{1}{\sigma^{2}}$ with q=48.

In Figure 7a,b the MSE of the GSP-WLS, pm-WLS, and mc estimators is presented versus the number of measured buses, *q*, and versus $\frac{1}{\sigma^{2}}$, respectively, with the sampling policies (i)–(iii). Figure 7b is obtained for q=48 measured buses, for which the system is not observable with probability 1. It can be seen that the MSE decreases as *q* increases and as $\sigma^{2}$ decreases. In both figures, the GSP-WLS estimator outperforms the pm-WLS and mc estimators for any tested sampling policy. In this case, the performance of the pm-WLS and the mc estimators is similar, since it can be shown that for the mc estimator, the rank regularization on θ (see Eq. (13a) in [5]) turns out to be an $l_{2}$ regularization on $\theta -{\hat{\theta }}_{prior}$, as in (29), where ${\hat{\theta }}_{prior}$ approaches zero. In addition, it can be seen that the proposed sampling policy from Algorithm 1 results in a significantly lower MSE than that obtained for the random and the E-design sampling policies for all estimators. In Figure 7a, it can be seen that for each sampling policy, the MSE of the GSP-WLS, pm-WLS, and mc estimators coincides with that of the other methods where the system becomes observable (i.e., where q>72 for the random sampling, q>76 for the E-design sampling, and q>79 for Algorithm 1). In particular, where q=118 (i.e., full observability with probability 1), the MSEs of all estimators are identical.

**Figure 8:**
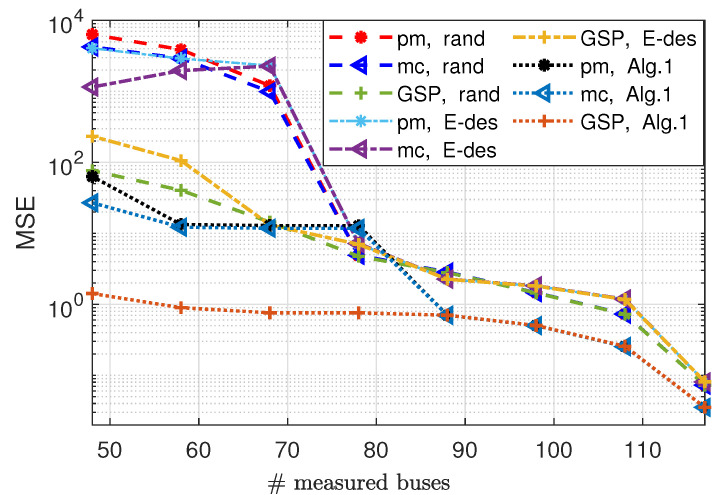
Power estimation based on the DC−PF model: the MSE of the GSP−WLS (26) and (27), pm−WLS [11], and mc [5] estimators for random, E−design [38], and Algorithm 1 bus selection policies versus the number of buses, *q*, where $\sigma^{2}=0.01$.

Figure 8 shows the MSE of the power estimator $\hat{z}$ from (33) with the three estimators and the three sampling policies. It can be seen that the MSE decreases as the number of measured buses increases, as expected, since there are fewer parameters to estimate with the increase in the number of samples and the state estimation is more accurate, as presented in Figure 7a. It can be seen that the relationships between the sampling policies and the estimators are similar to Figure 7a, where the GSP-WLS estimator with the bus selection policy of Algorithm 1 achieves the lowest MSE. Moreover, for each sampling policy, the MSE of the power estimation based on the GSP-WLS, pm-WLS, and mc estimators coincides with that of the other methods where the system becomes observable (i.e., where q>72 for the random sampling, q>76 for the E-design sampling, and q>79 for Algorithm 1).

**Figure 9:**
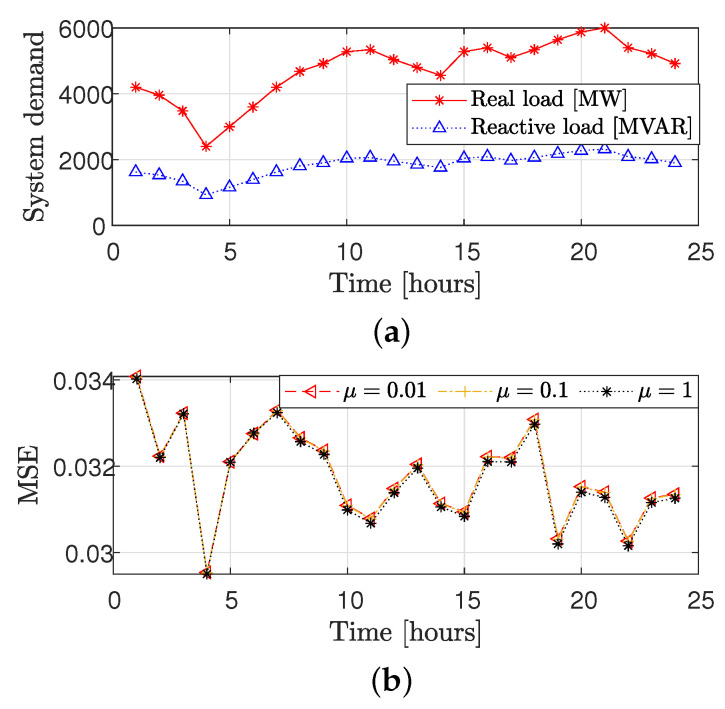
State estimation based on the DC−PF model: the hourly system demand (**a**) and the corresponding MSE of the GSP−WLS estimator (26) and (27) (**b**) versus time over 24 h for q=48 buses and $\sigma^{2}=0.01$.

In Table 2 and in Figure 9, the influence of the tuning parameter, μ, is examined; we show that the proposed GSP-WLS estimator is robust to the choice of this tuning parameter by demonstrating that the estimator achieves the same performance for a range of values of μ. In Table 2, the average and the sample standard deviation of the MSE of the GSP-WLS estimator for different values of μ∈[0.01,10] are presented, for a system with q=48 and $\sigma^{2}=0.01$, for the three sampling policies. It can be seen that, for the tested scenario, the MSE is approximately constant for any μ in the range [0.01,10]. Of course, further increasing μ will eventually increase the MSE, since the weight of the measurements in the estimation will be negligible. Similar results were obtained for other values of *q* and σ. Therefore, choosing any μ in this wide range results in good estimation performance for any sampling policy.

Figure 9 demonstrates the performance of the GSP-WLS estimator where the power system operating condition changes throughout the day, using time series data for different values of μ and correspondingly different sampling sets chosen by Algorithm 1. Simulation setup and parameters: For this case study, we use the modified IEEE 118-bus system in a similar manner to [67]. Figure 9a shows the hourly load profile of the system demand, as taken from http://motor.ece.iit.edu/Data/ (accessed on 1 August 2022). Given a single measure of the loads in all the buses and the total system demand along 24 h, the hourly load at bus *n* is the relative part of the load (among the other measured loads) from the total system demand. The true value of the state was calculated using the MATPOWER toolbox [68]. It can be seen in Figure 9b that for the MSE for μ=0.01,0.1,1 is approximately the same. These results show that the proposed GSP-WLS estimator is robust to the tuning parameter in this range, even under changing operating conditions. More strategies for choosing μ are described in the literature (e.g., see [61]).

### 6.3. State Estimation and Sampling under the AC-PF Model

In the following, we discuss the estimation of both the phases and the magnitudes of the states using the estimators:

(1) The Gauss–Newton implementation of the pm-WLS estimator from [11] by Algorithm 2 with the appropriate replacements of the regularization terms, i.e., where ${\hat{x}}_{prior}=x_{0}$ and $R_{prior}^{-1}=I$ are used instead of $\bar{L}\left(\mu_{\theta },\mu_{v}\right)$ (see the remarks after (57)).
(2) The mc method from [5], implemented using Equations (6), (8), (12a), and (12c) from [5], where the low-rank matrix used in this method, composed of the real and the imaginary parts of v. In addition, we added to this method the current measurements as inputs (instead of the power flow measurements) for a fair comparison. The implementation was conducted by the SDP solver of CVX [60].
(3) The proposed regularized Gauss–Newton method for implementing the GSP-WLS estimator from Algorithm 2, with the regularization parameters $\mu_{\theta }=0.045$ and $\mu_{v}=10$.

In the Gauss–Newton-based methods, the maximal number of iterations is set to l=20 and $\delta =10^{-8}$ in Algorithm 2.

**Figure 10:**
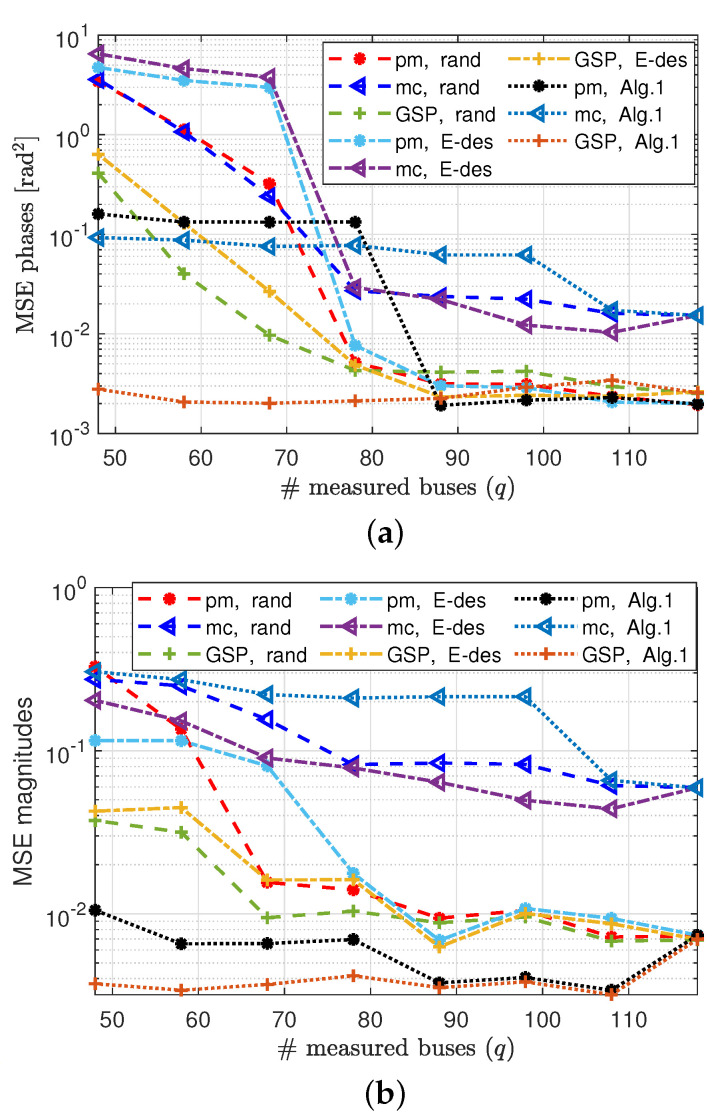
State estimation under the AC−PF model: the phase MSE (**a**) and the magnitude MSE (**b**) of the pm−WLS [11], mc [5], and GSP−WLS (Algorithm 2) estimators for random, E−design [38], and Algorithm 1 bus selection policies versus the number of buses, *q*.

In Figure 10a the MSE of phase estimation by the GSP-WLS and pm-WLS estimators (both implemented by the regularized Gauss–Newton method) are presented versus the number of measured buses, *q*, for the sampling policies (i)–(iii). Similarly, in Figure 10b, the MSE of the magnitude estimation is presented. It can be seen that the MSE decreases as the number of measured buses increases for both the magnitudes and the phases. Moreover, the GSP-WLS estimator outperforms the pm-WLS and the mc estimators for any sampling policy. Figure 10a shows that for each sampling policy, the MSEs of the GSP-WLS, and the pm-WLS estimators for the phases separate from each other when the system observability can no longer be guaranteed (i.e., where q<72 for the random sampling, q<76 for the E-design sampling, and q<79 for Algorithm 1). In particular, where q=118 (i.e., full observability with probability 1), the performances of the estimators coincide. Figure 10b demonstrates that for q=118, the MSEs of the GSP-WLS and the pm-WLS estimators for magnitude are equal. For an observable system with q<118, the MSEs of the estimators coincide for each sampling policy separately. Finally, when the system becomes unobservable (i.e., where q<72 for the random sampling, q<76 for the E-design sampling, and q<79 for Algorithm 1), the MSEs of the estimators split, and the MSE of the GSP-WLS is lower.

It should be noted that the mc estimator implementation by CVX has higher computational complexity and a lot of tuning parameters in comparison to the other methods. Finally, it can be seen that the sampling policy from Algorithm 1 results in a significantly lower MSE than that obtained for the random and the E-design sampling policies for the pm-WLS and the GSP-WLS estimators.

**Figure 11:**
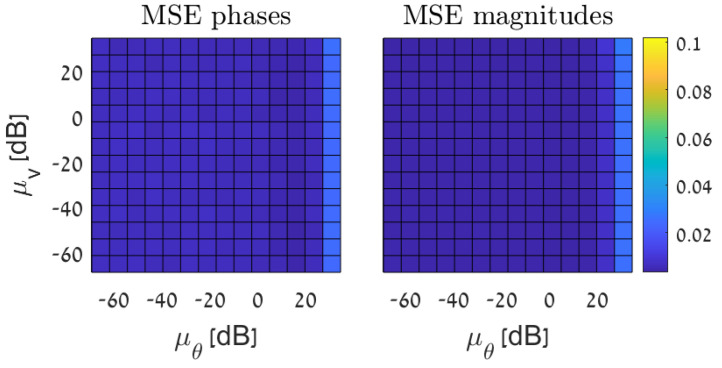
State estimation based on the AC−PF model: the MSE of the GSP−WLS (Algorithm 2) for phase estimation (**left**) and magnitude estimation (**right**) with q=48 buses and $\sigma^{2}=0.01$ versus the value of the tuning parameters, $\mu_{\theta }$ and $\mu_{v}$.

In Figure 11, we examine the influence of the tuning parameters $\mu_{\theta }$ and $\mu_{v}$ on the estimation performance of the phases and magnitudes, where the sampling set is chosen by Algorithm 1. It can be seen that for $\mu_{\theta },\mu_{v}\in \left[0.01,10\right]$, the MSE is approximately constant. Hence, choosing any $\mu_{\theta }$ and $\mu_{v}$ in this range will obtain a good result.

### 6.4. Detection of Bad Data

In the following, the application of bad data detection based on the proposed estimator is demonstrated, based on the description in Section 4.4. We compare the performance of the following detectors:

- The LNR test from (34), the J(θ) test from (35), and the GFT-based detector from [7], after substituting either $\hat{\theta }={\hat{\theta }}^{\mathrm{pm}-\mathrm{WLS}}$ from (29) or $\hat{\theta }={\hat{\theta }}^{\mathrm{GSP}-\mathrm{WLS}}$ from (26) and (27). In the GFT-based method, the cutoff frequency is chosen such that in normal states, 35% of the frequencies pass the filter.
- The $L_{\infty }$ detector from [69] with $\Sigma_{x}=I$.
- The Laplacian-Regularized detector (Lap.Reg.) from our previous work in [41], which also uses the GSP-WLS estimator, ${\hat{\theta }}^{\mathrm{GSP}-\mathrm{WLS}}$.

Simulation setup and parameters: The detection performance is evaluated for partially observable systems with only power injection measurements, i.e., $L_{\nu ,S}=H_{\nu ,S}$, with q=48 buses that are chosen by Algorithm 1. Constant noise (Δz=10σ) has been added onto three random measurements, i.e., $z_{i}+\Delta z$ at the *i*th bus, where $z_{i}$ is obtained by (21).

**Figure 12:**
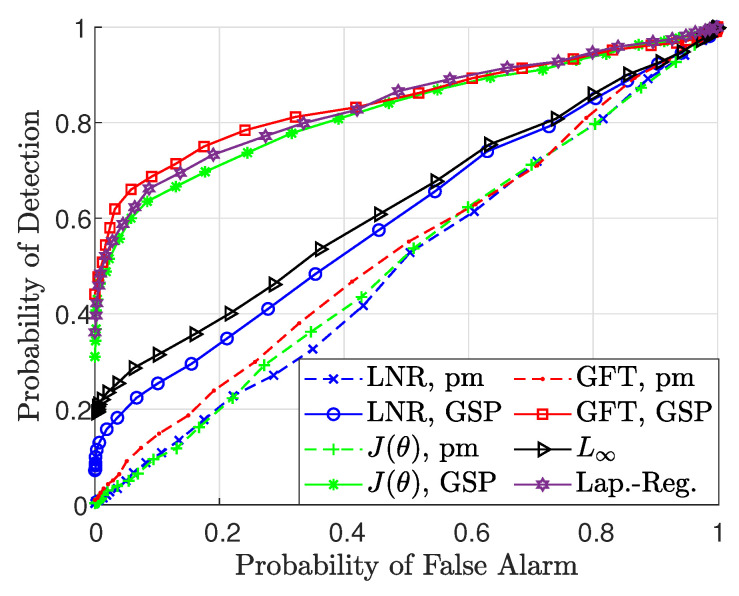
Bad data detection: the ROC of the LNR test (34), J(θ) test (35), GFT−based detector [7] implemented with the GSP−WLS (26) and (27), and the pm−WLS [11] estimators, $L_{\infty }$ [69], and the Laplacian−regularized [41] detectors with q=48 buses.

In Figure 12, the detection performance of the proposed detectors is demonstrated by the receiver operating characteristic (ROC) curves. This figure shows that the detectors that are based on the GSP-WLS estimator outperform the detectors that are based on the pm-WLS estimator, as well as the $L_{\infty }$ detector. These results are aligned with the estimation results, since the MSE of the state estimation by the GSP-WLS estimator is significantly lower than the MSE obtained by the pm-WLS estimator, as shown in Figure 7.

## 7. Discussion

Utilization and Implications: The proposed GSP-WLS estimator has the potential to significantly improve state estimation in unobservable power systems, which can be caused by various reasons, such as communication issues, cyber attacks, and legal or economic constraints on measurement locations. By utilizing GSP techniques, the proposed estimator can achieve more accurate estimates with fewer measurements, reducing the cost and complexity of data collection. In addition, the proposed sensor placement strategy optimizes the performance of the estimator, further improving its accuracy. These GSP techniques have important implications for the reliability and stability of power systems, as well as for the development of advanced control and optimization algorithms. The research also demonstrates the capabilities of GSP in power system networks, highlighting the potential for its use in other tasks within the field. Overall, this research aims to increase the stability and reliability of electrical grids by developing monitoring techniques. Additionally, this study is closely related to the control and management of renewable energy sources, since using enhanced estimation, sensor allocation, and attack detection methods, the ability of the grid to deal with randomness in the system that stems from the sources increases.

Limitations: This research provides a new GSP framework for state estimation and bad data detection. However, it has some limitations that should be considered in future research. First, it only uses the most recent vector of measurements. While this is a good policy when the system may be under abrupt changes, the performance of the estimator can be improved by incorporating historical measurements from previous time steps. Second, the topology of the network is assumed to be known, which may not always be the case, particularly for distribution systems. In such cases, methods for estimating the topology [29,70] could be used. Third, the Gauss–Newton algorithm used in this study may be computationally expensive for large networks. To address this issue, future research could use low-complexity algorithms to calculate matrix inversion, as described in [1]. Finally, this study has not explored the incorporation of smart meter and PMU measurements into the proposed methods, and this should be considered in future research.

Future research: Future research directions include the incorporation of time series measurements with temporal dependencies to improve the estimation and bad data performance. Additionally, it would be useful to investigate the joint estimation of the system state and the identification of topology changes. Another potential future direction is the combination of smart meter measurements and PMU data in the proposed method, as mentioned above in the limitations. Finally, a promising area of future research is the integration of deep unfolding or other machine learning and optimization tools to accelerate the regularized Gauss–Newton method. One particularly important area of future research is the development of new graph neural network (GNN) approaches that take advantage of the graph properties of the states that were validated in this study. GNN methods could potentially offer significant improvements in the accuracy and efficiency of state estimation in power systems, depending on the availability of relevant data.

## 8. Conclusions

This paper proposes a GSP framework for state estimation, sensor allocation, and bad data detection in unobservable power systems. First, the graph smoothness of the phases and the magnitudes of the voltages with respect to the admittance matrix have been validated. Then, the GSP-WLS estimator of the system states has been derived under both the DC-PF and AC-PF models. The GSP-WLS estimator uses the graph smoothness of the state signals as a regularization term, and thus, does not require full observability of the system. It is analytically shown that when the system is observable, the proposed GSP-WLS estimator coincides with the WLS estimator. A greedy algorithm has been introduced to tackle the problem of selecting the sampling set that optimizes the state estimation performance. In addition, it is shown that the GSP-WLS estimator is useful for bad data detection by plugging the estimator into existing detectors. Simulation results demonstrate the potential of the GSP methods in power systems for cases where the system observability is not guaranteed. It is shown that the proposed methods can accurately estimate voltage phases and magnitudes, and detect bad data under partial observability conditions where standard methods cannot, or exhibit poor performance. These results show that advanced sensing and measurement technologies using GSP tools can transform data into useful information and enhance various aspects of power system management.

## Figures and Tables

**Table 1:** Normalized smoothness values of IEEE systems.

Measure	IEEE Test Case System				
	14-Bus	30-Bus	57-Bus	118-Bus	300-Bus
$\frac{E_{L}\left(\theta \right)}{{\left||\theta |\right|}^{2}}$	0.6617	0.3015	0.3714	1.1740	1.2371
$\frac{E_{L}\left(v\right)}{{\left||v|\right|}^{2}}$	0.0036	0.0022	0.008	0.0082	0.0199
$\frac{E_{L}\left(z_{\mathrm{bus}}\right)}{\left|\right|z_{\mathrm{bus}}{\left|\right|}^{2}}$	16.4079	18.3307	50.8035	56.1047	138.8024

**Table 2:** State estimation based on the DC-PF model: the average MSE and its standard deviation (std.) for the GSP-WLS (26) and (27) over different values of μ∈[0.01,1], for q=48 and $\sigma^{2}=0.01$.

Measure	Bus Selection Method		
	Random	E-Design	Algorithm 1
Average MSE	0.2479	0.7929	0.0116
std. MSE	0.0116	0.0701	$9.45\times 10^{-6}$

## Data Availability

The IEEE test case systems data in the simulations has been taken from the “Power Systems Test Case Archive”, ref. [56], http://www.ee.washington.edu/research/pstca/ (accessed on 1 June 2019).

## Abbreviations

The following abbreviations are used in this manuscript:

GSP	Graph signal processing
WLS	Weighted least squares
PF	Power flow
DC	Direct current
AC	Alternating current
PSSE	Power systems state estimation
EMS	Energy management system
GFT	Graph Fourier transform
DSP	Digital signal processing
KKT	Karush–Kuhn–Tucker
pm	Pseudo-measurements
MSE	Mean squared error
CRB	Cram$\overset{´}{e}$r–Rao bound
LNR	Largest normalized residual
FDI	False data injection
ROC	Receiver operating characteristic
GNN	Graph neural network
